# Group exercise improves chronic pain in South Florida older adults

**DOI:** 10.1186/1753-6561-6-S4-O51

**Published:** 2012-07-09

**Authors:** N Kapila, H Florez, S Dang, L Oropesa

**Affiliations:** 1 Royal College of Surgeons in Ireland; 2 University of Miami, USA

## Introduction

Chronic pain in older adults is a serious and debilitating issue, often overlooked and mismanaged by primary care physicians. Chronic pain affects 25-50% of community dwelling older adults, and 45-80% of older adults residing in long-term care facilities. Exercise in geriatric populations is necessary to maintain mobility and quality of life. We analyzed 129 Veterans at the Miami VA Medical Center who were enrolled in an evidence based exercise program. The objective of this study was to determine the impact of an evidence-based exercise program on chronic pain in the older adults of South Florida.

## Methods

Quasi-experimental study in a community-based Veterans Affairs Healthcare System. Adults (n=129), ages 66.5 ± 5.6 y, with Body Mass Index (BMI) of 34.8 ± 6.2. 98.5% male were enrolled in the MOVE! Weight Management Program. Participants were divided into two groups: Good Adherence (GA=55) and Poor Adherence (PA=74) where GA ≥ 50 and PA < 50, according to class attendance, a program consisting of 1-hour sessions three times per week. Pain was assessed using the Brief Pain Inventory (BPI), a 16 question survey scoring Pain Interference (PI) and Pain Severity (PS). Data was collected at baseline, 4 months, and 1 year and analyzed using covariance.

## Results

The GA and PA groups had similar demographic, anthropometric, metabolic and pain profiles at baseline. After four months, the GA group achieved greater improvements in PI (3.2 ± 2.7, p = 0.03). Moreover, age was a significant factor influencing improvements on PS change. After four months, the GA group had considerable improvement in physical function using the Hand Grip (HG) (3.8 ± 0.9, p = 0.004). After one year changes were significant where the GA group had improvement in HG (8.4 ± 1.4, p = 0.007). After one year, the GA group noted less fatigue (-13.8 ± 3.7, p = 0.006) than initially reported.

## Conclusions

The data suggests that good adherence to an exercise program is an integral component for pain relief and management, physical function, and fatigue. The data presented can lay a foundation for future improvements in pain management for the geriatric population.

